# Variations in attitudes towards stereotactic biopsy of adult diffuse midline glioma patients: a survey of members of the AANS/CNS Tumor Section

**DOI:** 10.1007/s11060-020-03585-7

**Published:** 2020-07-23

**Authors:** John Lynes, Alvina A. Acquaye, Hannah Sur, Anthony Nwankwo, Victoria Sanchez, Elizabeth Vera, Tianxia Wu, Brett Theeler, Terri S. Armstrong, Mark R. Gilbert, Edjah K. Nduom

**Affiliations:** 1 Surgical Neurology Branch, National Institute of Neurological Disorders and Stroke, National Institutes of Health (NIH), 10 Center Drive, Room 3D20, Bethesda, MD 20892 USA; 2 Neuro-Oncology Branch, National Cancer Institute, National Institutes of Health, Bethesda, MD USA; 3 Clinical Trials Unit, National Institute of Neurological Disorders and Stroke, National Institutes of Health, Bethesda, MD USA

**Keywords:** Adult diffuse midline glioma, Glioma, Brain neoplasm, Biopsy

## Abstract

**Purpose:**

Diffuse midline gliomas are rare midline CNS malignancies that primarily affect children but can also affect adults. While radiation is standard treatment, prognosis remains fatal. Furthermore, due to its sensitive anatomic location, many physicians have been reluctant to perform biopsies without potential for improved prognosis. However, recent advancements in molecular-targeted therapeutics have encouraged greater tissue sampling. While the literature reflects this progress, the landscape of how clinicians actually manage these patients remains unclear. Our goal was to assess the attitudes of current practicing neurosurgical oncologists towards management of adult diffuse midline gliomas, reasons behind their practices, and factors that might influence these practices.

**Methods:**

We created and distributed a survey with 16 multiple choice and open-ended questions to members of the Tumor Section of the Congress of Neurological Surgeons.

**Results:**

A total of 81 physicians responded to the survey. Although time since training and volume of glioma patients did not significantly affect the decision to consider clinical trials or to offer biopsy, those that operated on fewer gliomas (< 25/year) were more likely to cite surgical morbidity as the primary reason not to biopsy these midline locations. Further, surgeons with access to more advanced molecular testing were significantly more likely to consider clinical trial eligibility when offering biopsies.

**Conclusion:**

Factors that affect the management of diffuse midline gliomas and the role of biopsy are relatively uniform across the field, however, there were a few notable differences that reflect the changes within the neuro-oncology field in response to clinical trials.

**Electronic supplementary material:**

The online version of this article (10.1007/s11060-020-03585-7) contains supplementary material, which is available to authorized users.

## Introduction

Diffuse midline gliomas (DMG) are a group of high-grade neoplasms that arise from CNS midline locations [1]. These lesions are nearly uniformly fatal, and new treatment modalities are needed [2]. However, the advancement of the field has been limited by the lack of molecular understanding of these lesions [3]. While progress has been made in the understanding of gliomagenesis of these lesions in both children and adults, tissue sampling is still not routine practice for these cases, although biopsies are more common in pediatrics.

Although DMGs are more prevalent in pediatric patients [4], diffuse midline gliomas are also found in adults [5, 6]. According to guidelines, the accepted standard treatment for these lesions is radiation [7], as surgical resection is severely limited by their critical locations, and chemotherapy has typically been found to be ineffective [8, 9]. Moreover, these tumors have typically been diagnosed using MR imaging alone, foregoing the necessity of diagnostic biopsy [10, 11]. Still, the prognosis for patients with DMG remains very poor with an average survival time of only one year following diagnosis [12].

Recent advancements in the field have discovered relevant molecular mutations in DMGs [13, 14], giving way to broad developments in diagnosis and treatment of these tumors. We now have reason to biopsy these patients to develop targeted therapeutics [15] and clinical trials to test them. However, despite this progress, the standard practice for diagnosing and treating this disease is unclear, and management can vary across clinical sites. We sought to describe the current range of practice by practicing neurosurgeons. Our survey assessed the attitudes of neurosurgical oncologists who are members of the Joint Tumor Section of the American Association of Neurological Surgeons (AANS) and Congress of Neurological Surgeons (CNS) regarding tissue sampling of DMGs. Our aim was to define their current decision making in the management of DMGs, reasoning for their practices, and potential factors that may influence or change their practice.

## Materials and methods

### Survey design and distribution

Our survey was designed and reported in accordance with published guidelines [16]. An open and voluntary survey consisting of 16 multiple-choice questions (see Electronic Supplemental Material for full survey) was developed by a multi-disciplinary group of expert providers, including representatives from the Surgical Neurology Branch (SNB) of NINDS and the NCI-CONNECT program of the Neuro-Oncology Branch (NOB), CCR, NCI, NIH. It was also vetted by the Tumor Section Survey Committee prior to approval. This research survey was excluded from IRB Review per 45 CFR 46 and NIH policy (OHSRP ID#: 19-NINDS-00813) for the use of survey procedures, because it involved the use of survey procedures and the recorded information was not linked to identifiable human subjects, directly or through identifiers linked to the subjects. The survey was web-based and sent via an e-mailed link to the 636 active members of the Joint Tumor Section of the AANS and CNS listserve and advertised via the official Twitter account of the Joint Tumor Section (@NSTumorSection). The Tumor Section is a voluntary, membership-only group of practicing neurosurgeons who specialize in tumors of the central nervous system. Responses were collected for 4 weeks (from 10/2/19 to 10/30/19). It was created on Surveymonkey.com with allowance for custom answers to specific questions for respondents if they believed the choices provided did not accurately reflect their response. As some questions depended on answers to previous questions, the order of the questions was not randomized, and adaptive questioning was employed. Any user with the survey link was free to participate; no incentives were offered. All of the answers were automatically captured by the secure SurveyMonkey database and exported to an excel and PDF file for analysis. To prevent multiple entries from the same device, IP addresses were collected, and duplicate entries were ‘turned off’ for the survey, thus only allowing one survey response per device. These IP addresses could not be traced to an individual person, but rather to a single device, proxy server, or group of devices on the same network. Responses for each question were grouped based on potential significance and/or frequency that a particular answer was chosen. These groups were then compared to surgeon and institution-specific characteristics in order to assess for correlations.

### Statistical analysis

A one-way analysis of variance (ANOVA) was performed to evaluate differences in years of experience between surgeons who offered and did not offer biopsies for a diffuse glioma (questionnaire 6,10, 11,13, 14 and 16). The experience years were calculated from the date neurosurgical residency training was completed to the date the survey was completed. Fisher’s exact test was applied to test the association between the number of operated patients in the past 12 months (Q4) and the willingness to offer biopsy at various midline locations (Q6, Q10, Q11, Q13, Q14, Q16). The number of operated patients was dichotomized into a binary variable: ≤ 25 and > 25. Fisher’s exact test was also applied to evaluate the association between the reason of offering biopsy (Q13) and treatment decision (Q15). SAS software 9.4 was used for statistical analyses. A statistical significance level was set at α = 0.05.

### Qualitative analysis

**Fig. 1:**
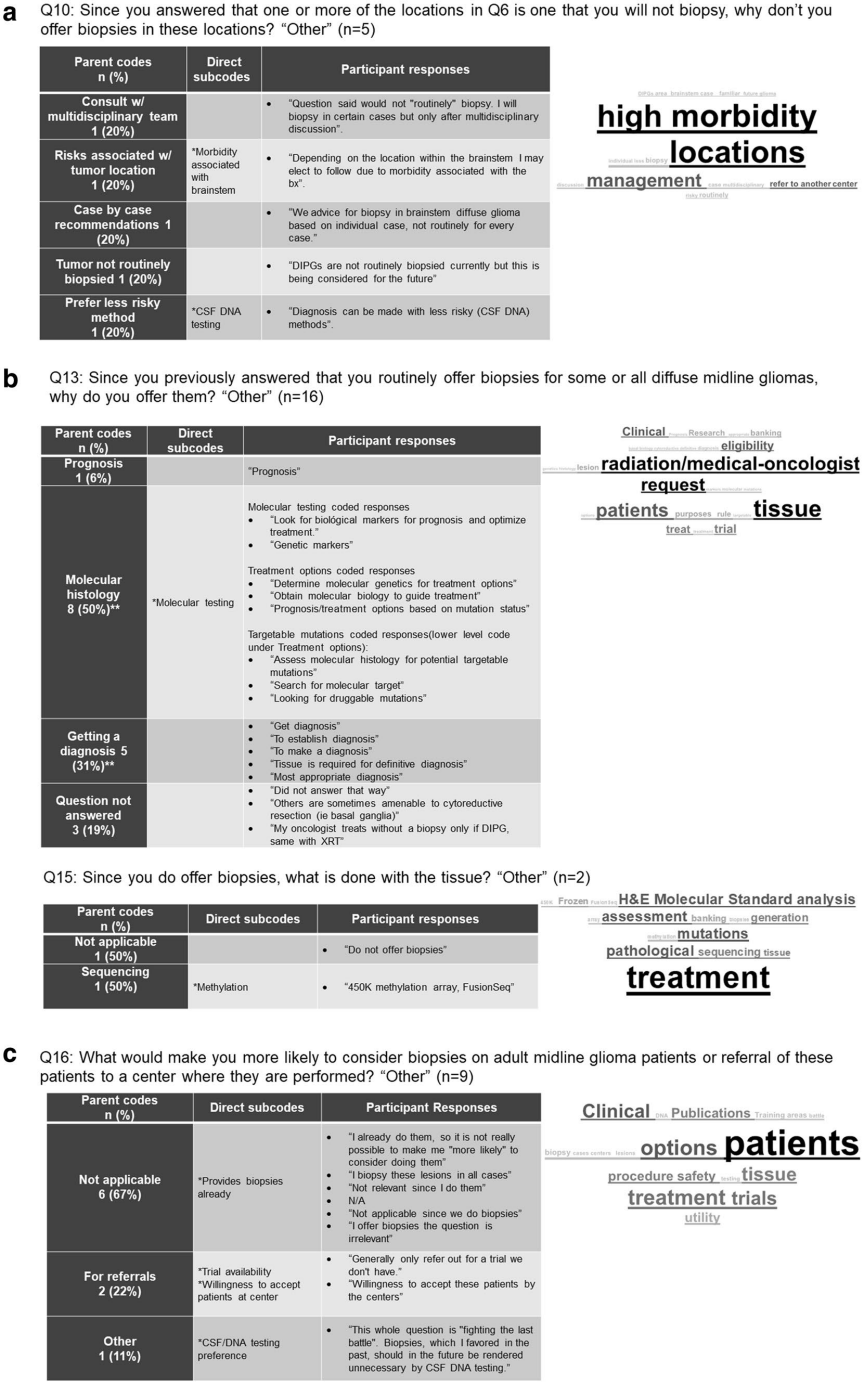
Qualitative analysis of the open-ended answers that respondents entered if they chose “other” to a question. The associated word clouds depict which key words were most frequently written. **a** Qualitative analysis of free-text answers to question 10, which follows respondents who selected one or more midline locations as those that they would not offer biopsy. **b** Qualitative analysis of free-text answers to question 13 and 15, which follows respondents who said they do offer routine biopsy of diffuse midline gliomas and asks why they offer them and what they do with the tissue. **c** Qualitative analysis of free-text answers to question 16, which asks all respondents what would potentially increase the likelihood of offering biopsy to adult DMG patients or referral to other centers where biopsy of these locations is offered

Given that four of the questions (Q10, Q13, Q15, Q16) included the ability to answer “Other” and give a personalized response, qualitative analysis of these responses was performed using MAXQDA 2019 software (VERBI Software, Berlin, Germany). Responses were then categorized into parent codes and direct subcodes. Additionally, for these four questions, word clouds were created from survey respondent answers (Fig. 1a–c).

## Results

### Characteristics of survey respondents

**Fig. 2:**
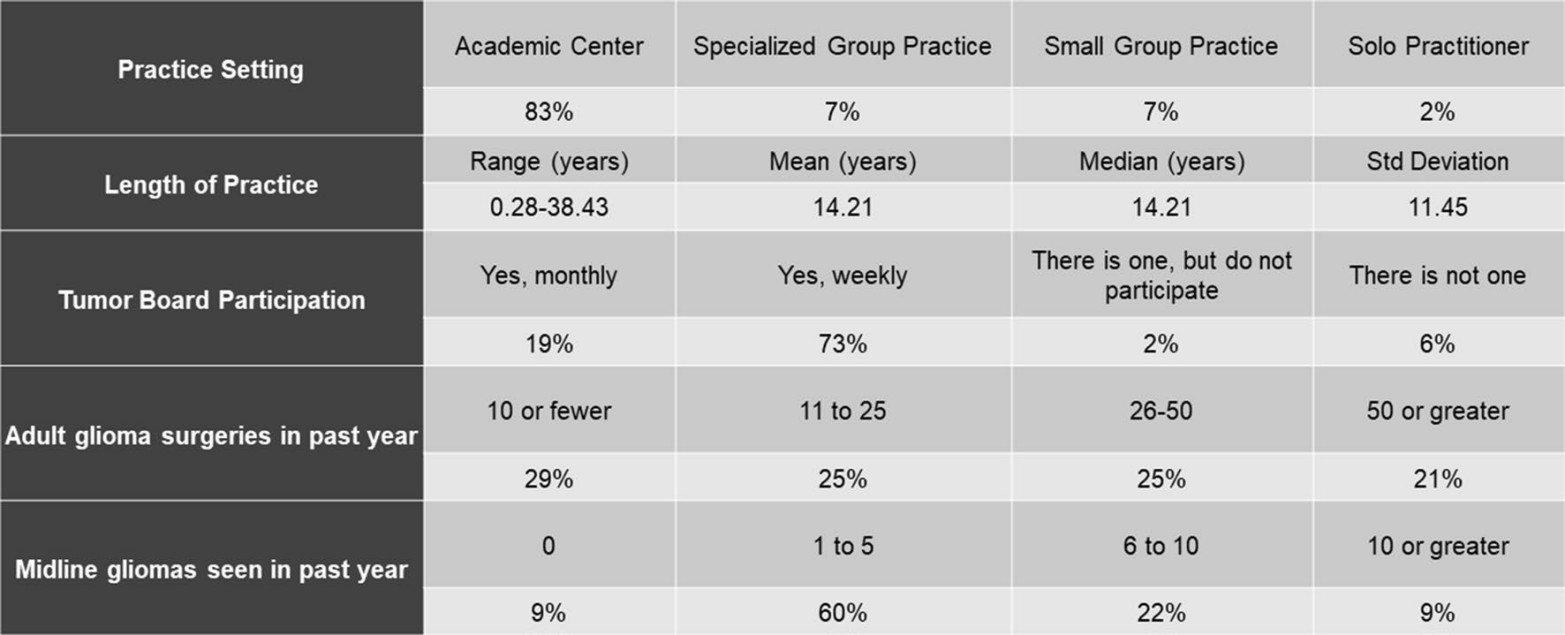
Respondent demographics. This breaks down the respondents practice setting, length of practice, frequency of tumor board participation, volume of adult glioma surgeries in the past 12 months, and volume of midline glioma patients seen in the past 12 months

A total of 81 neurosurgeons completed the survey out of 636 active members of the Tumor Section of CNS. However, a wide range in the number of responses were submitted for each of the 16 questions (range 2–81), as answering all questions was not required for submission and the survey logic presented different questions based off of answers to previous questions from question 7 onwards (Electronic Supplemental Material). Respondents included surgeons with varied training backgrounds and practices, which provided a broad view of surgeon and institution-specific characteristics as well as management strategies. A large majority (83%) of respondents practice in academic centers, and 91% participate in tumor boards at their institutions (Fig. 2). 90% of surgeons had some form of molecular analysis available at their institutions, though more advanced next-generation sequencing was only available to 60% of these. Surgeons ranged widely in their time in practice, from approximately 4 months to over 38 years with a mean of 14.21 years.

Less than half of the surgeons surveyed (41%) routinely consider biopsies of DMGs regardless of their anatomic location. Among the respondents who selectively biopsy certain midline locations, the brainstem was the most common response (51%) for the anatomic location that they do not routinely biopsy. Only 11% of surgeons reported reluctance to offer biopsies within the basal ganglia or thalamus. Of those who report reluctance of biopsies in particular anatomic locations, 70% report that procedural morbidity was a concern. 37% report that they do not routinely biopsy in these locations because biopsy would not change their management decisions. In ranking these concerns, 58% indicate morbidity is their greatest reason, while 24% report that tumor sampling not changing management of the patient is their largest concern.

### Reasons for offering biopsy

Qualitative analysis of open-ended responses demonstrated that surgeons offered biopsy in order to obtain molecular histology and diagnosis (50 and 31% of open-ended responses, respectively) (Fig. 1b). Also, 50% (38) of surgeons who responded to this question reported performing biopsies due to requests from radiation oncology colleagues, and 46% (35) perform biopsies due to requests by medical oncologists prior to initiating therapy. Surgeons who do not offer biopsies are much more likely to refer patients directly to a neuro-oncologist, radiation oncologist, or both at their institution (40, 89%) than to another institution (5, 11%).

### Factors that affect the likelihood of offering a biopsy

Fisher’s exact test indicated that the reason for offering biopsies was associated with the method for evaluating the samples (*p* = 0.003). Surgeons who only have access to conventional histopathology (i.e. H&E stained slides) (7%) are less likely to indicate clinical trial eligibility as a factor or as the most important factor in whether or not to offer biopsy. Conversely, surgeons who have molecular testing or sequencing available were more likely to indicate clinical trial eligibility as the most important factor in their decision to offer a biopsy (*p* = 0.003). While 55% of surgeons who offer biopsies consider clinical trial eligibility in the management of these patients, only 21% report that it is the most important indication for biopsy. Qualitative analysis demonstrated that a subset of surgeons added that the decision to biopsy is nuanced, with 20% reporting that the decision is based on consensus of a multidisciplinary team, and 20% reporting that the decision is made on a case-by-case basis (Fig. 1a). Additionally, 22% of custom responses indicated that availability of trials at other institutions and willingness of other centers to accept these patients would increase their likelihood of offering biopsies (Fig. 1c).

### Time since training and likelihood to offer a biopsy

No significant differences were noted in management of midline gliomas based on length of time since residency training. There were no significant differences found for time since training that dictated whether or not biopsies would be offered in particular anatomic locations (*p* = 0.78), whether morbidity is the greatest concern for not offering biopsies (*p* = 0.49), or whether clinical trial eligibility was a factor in offering biopsies (*p* = 0.92). Similarly, there were no significant differences correlating time since training with clinical trial eligibility or medical/radiation oncological need for tissue as the most important consideration (*p* = 0.59). Lastly, safety of biopsy and new treatment options were not factors that changed consideration of biopsy across differing times since residency training (*p* = 0.93).

### Volume of glioma patients seen and likelihood to offer a biopsy

The minority (29%) of the neurosurgeons surveyed operated on fewer than 10 total glioma patients annually, and 9% had not operated on any midline gliomas in the past 12 months. Conversely, 21% of surgeons operated on more than 50 glioma patients and 9% had operated on more than 10 midline glioma patients in the previous 12 months. Surgeons who operated on fewer gliomas (< 25 patients, 53.75% of survey respondents) in the last 12 months were similarly likely to offer biopsies regardless of anatomic location (*p* = 0.90) or to consider clinical trial enrollment as an indication for biopsy (*p* = 0.82). Also, these surgeons are not more or less likely to indicate demonstrated safety, additional training, or new treatment options as reasons to consider biopsies in more midline glioma patients (*p* = 1.00). However, these same surgeons who operated on fewer gliomas in the previous year (< 25 glioma patients a year, 54% of respondents) were significantly more likely to cite surgical morbidity as their most important reason for not offering biopsies in particular anatomic locations (*p* = 0.04). We found that this concern does not appear to reflect a discomfort or unfamiliarity with performing the procedure, as only 12% felt that additional training in safe biopsy of these sensitive areas would increase their likelihood of offering biopsy.

## Discussion

We developed a survey to distribute to neuro-oncologic surgeons who are members of the Tumor Section of the Congress of Neurological Surgeons. This survey was designed to elaborate surgeon demographics, institutional characteristics, and clinical practice in the management of adult midline gliomas. Our survey’s implications are potentially limited because a significant majority of respondents practice within an academic institution, and therefore it may not be reflective of general neurosurgery practice. However, many patients diagnosed with this rare disease may be referred or self-refer to academic institutions for management. With less than 500 diagnoses of the adult form of this disease made per year in the United States [17], diffuse midline glioma is an extremely rare disease, as reflected by 9% of respondents not having managed an adult midline glioma in the previous 12 months.

The willingness to biopsy midline glioma patients has a direct bearing on our ability to understand their biology. Based on samples from these lesions, the histone H3 K27M somatic mutation has been identified in pediatric and adult tumors [13, 18]. This has led to a new WHO 2016 classification of “diffuse midline glioma, H3 K27M-mutant” [19]. However, the prevalence and significance of this mutation remains controversial. Schreck et al. [20] reported the presence of H3K27M mutations in 18 of 123 cases (15%) of the adult midline glioma cases studied, with locations in the midbrain, pons, and cerebellum. They also found that patients with this molecular profile had improved median survival of 17.6 months over 7.7-month survival of patients with high-grade wildtype tumors, p = 0.03. Conversely, Ebrahimi et al. [21] reported a higher rate of the mutation, 24%, in their cohort of adult tumors, but found no difference in overall survival for these patients. Differences have also been reported between pediatric and adult forms of the disease [22]. However, another center’s experience reflects that H3 K27M mutation portends poor prognosis in both adult and pediatric midline gliomas irrespective of histologic features traditionally utilized for diagnosis and management [5]. The variability of reported work on these lesions suggests that greater molecular understanding is necessary. We will not be able to further expand this knowledge without neurosurgeons willing to biopsy these lesions.

In the context of improved understanding of the disease from tissue analysis, different factors correlate with the tendency to sample tissue. Institutional resources and limitations in biopsy practice appear to significantly affect surgeon practices. Surgeons without molecular testing or sequencing available were significantly less likely to consider clinical trial eligibility. As such, because non-academic centers likely have significantly less access to advanced tissue processing and analysis, it is possible that surgeons outside of academia, who are underrepresented in this survey’s respondents, are potentially even less likely to consider clinical trial enrollment.

Overall, we found that surgeon-specific experiences are not particularly influential to patient management. Among the neurosurgeons surveyed, we found a large proportion who would biopsy in any typical location of midline gliomas. There were also no differences based on years in practice since training in their overall willingness to perform midline glioma biopsies. Additionally, this factor and glioma patient volume had no significant impact on the way DMG is managed among surgeons.

We further sought to determine what guiding factors surgeons considered when contemplating whether or not to biopsy a midline glioma. The risk of surgical morbidity was the greatest concern respondents reported in not offering biopsies in particular anatomic locations, particularly among surgeons with a low frequency of glioma. However, this survey did not evaluate surgical outcomes among this population to elucidate whether this concern is valid. Published series suggest that this concern may be disproportionate and report relative safety of brainstem biopsy [23–25]. Studies have reported 0.4–2.2% long term morbidity and 0–1.46% mortality [26–28] with a large metanalysis reporting 0.6% permanent morbidity with 0.6% mortality [29]. This is very similar to the overall reported risk of morbidity in stereotactic biopsy in all locations [30]. Furthermore, multiple metanalyses reiterate the low morbidity of these procedures with different surgical approaches, frameless vs frame stereotactic technique, or use of robotic assistance [31]. In spite of these recent findings, 40% of our survey respondents report that publications demonstrating safety and utility of the procedure would make them more likely to consider biopsies of suspected midline gliomas. These findings may indicate a lack of familiarity with published literature by some of the study respondents, which further supports a need to more widely disseminate these published reports.

The second most common concern among surveyed surgeons was that biopsies will not change management of midline gliomas. However, the discovery of the various histone mutations associated with diffuse midline gliomas and our greater understanding of gliomagenesis have created new treatment options for these lesions [32, 33]. Clinical trials investigating the efficacy of targeted therapies against H3 K27M-mutant DMG are ongoing. There are dozens of ongoing early-phase clinical trials [34] investigating different treatments, both conventional and novel. For example, Gojo et al. report a small prospective study in which 10 patients’ tumors underwent comprehensive molecular analysis and 90% underwent personalized treatment based on identified targetable mutations. While no overall significant survival benefit was conferred over the control cohort, only the experimental cohort had long term survivors [35]. Additionally, ONC201, a dopamine receptor D2/3 (DRD2/3) antagonist, has demonstrated clinical activity in DMG patients with the H3K27M mutation [36]. In recent years, a large number of clinical trials have commenced investigating novel treatment for this disease, often necessitating molecular pathology information for study enrollment [37, 38]. Advancements in preclinical data are driving acceleration in clinical trials. Numerous molecularly targeted therapeutics have been identified for DMG, including treatments targeting epigenetic modification, growth factor receptors, signal transduction pathways, cell cycle checkpoints, stem cell signaling pathways, and DNA damage repair [39]. Lin et al. report use of high throughput drug screening of 2706 agents with 860 mechanisms of action on six H3K27M-mutated DMG cell culture models. Analysis of potency and blood–brain barrier penetration identified both the HDAC inhibitor, panobinostat, and the proteasome inhibitor, marizomib, as candidates for clinical use. They then demonstrate significant survival improvement in multiple murine xenograft models with this treatment combination [40].

Panobinostat and marizomib will be used in an upcoming phase 1 trial of pediatric patients with DIPG (NCT04341311) [41]. Meanwhile, two phase 1 trials investigating panobinostat administration via intratumoral convection-enhanced delivery are currently underway (NCT04264143, NCT03566199) [42, 43]. An ongoing phase 1 trial for another identified mutation in H3K27M DIPGs, B7-H3, will have an arm targeting these tumors via intraventricular infusion of targeted CAR T cells (NCT04185038) [44]. These ongoing clinical trial efforts may cause a paradigm shift requiring a diagnostic biopsy to rule-in the possibility of clinical trial enrollment. Notably, 79 and 67% of surgeons report new treatment options and clinical trials requiring tissue, respectively, would increase their likelihood to offer biopsy. Thus, there appears to be a discrepancy between current prevalence of potential clinical trials, particularly those investigating new treatment options, and surgeon knowledge of these trials. Part of this discrepancy may be a function of surgeons’ predilection to manage midline glioma patients once they have presented at their own institution, as only 11.11% report considering referral to another clinical center. As such, there may be an element of compartmentalization of the field, with consideration of biopsy for trial enrollment being a function of which studies are conducted at or affiliated with a surgeon’s own or familiar institutions.

While recent advancements in the understanding of the pathology of diffuse midline gliomas and identification of targetable pathways have been dependent on tissue sampling, groups are currently investigating noninvasive methods for evaluating mutational status in these tumors via imaging [45, 46] or sequencing of circulating tumor DNA [47, 48]. However, biopsy remains the gold standard for clinical trial consideration. Pending a change in this paradigm, we believe stereotactic biopsy should be considered at the time of initial presentation for all diffuse midline gliomas to maximize the opportunity for patients to participate in ongoing clinical trials, whenever clinical trial enrollment is possible and available for the patient. This burden must be shared by the entire neuro-oncology community, including neurosurgeons, neuro-oncologists, radiation oncologists, and other allied specialties to both develop and promote clinical trials for DMGs. Patient and caregiver engagement through programs like NCI-CONNNECT [49] are also important to inform both patients and their physicians about ongoing clinical trial efforts for patients with rare CNS cancers.

Though the results of the survey suggest trends of management of diffuse midline gliomas amongst neurosurgeons, there are limitations to this study. First, surveys, broadly, are subject to many inherent limitations, including recall bias, nonresponse bias, and low response rates [50]. Our survey questions, in attempting to steer respondents to limited specific responses, led to the generation of several “other” responses, which are captured in Figs. 1 and 2. Also, the survey questions regarding the number of surgeries performed in the preceding year (Questions 4 and 5) pooled responses to allow statistical comparisons to be made between the groups. Approximately half of respondents fell on each side of the chosen cutoffs, though these parameters are not direct indicators of technical mastery. Additionally, the survey was sent to members of the Joint Tumor Section of the American Association of Neurological Surgeons and Congress of Neurological Surgeons, a limited subset of practicing neurosurgeons. Nonetheless, given the rarity of diffuse midline gliomas, and given that neurosurgical oncologists would be expected to have the most experience with these lesions, we believe that our respondents likely skew towards a population of neurosurgeons who are more likely to biopsy than not. This makes the considerable percentage of neurosurgeons surveyed who do not routinely biopsy these lesions even more notable. Finally, the number of survey responses, eighty-one, limits the generalizability of the results. However, recently published surveys of neurosurgical oncologists have comparable response rates, so we feel this is a limitation of surveying this particular neurosurgeon population rather than our specific survey design [51–53].

## Conclusion

The authors surveyed surgeons within the Tumor Section of the Congress of Neurological Surgeons regarding their background, practices in management of midline glioma patients, and institutional characteristics. While there was notable similarity between care providers in the field, there were important differences between practitioners in the field regarding the role of biopsies and consideration of clinical trials. These differences may reflect the changes occurring within the field of midline glioma management in response to ongoing and future trials.

## Electronic supplementary material

Below is the link to the electronic supplementary material.

Supplementary file1 (PDF 773 kb)

Supplementary file2 (DOCX 28 kb)

## Data Availability

Authors declare that all data supporting the findings of this study are available within the article or its supplementary file, and available from the corresponding author upon request.

## Code availability

SAS software 9.4 was used for statistical analyses. MAXQDA 2019 software was used for qualitative analysis.
