# Supplementary material for: Variations in attitudes towards stereotactic biopsy of adult diffuse midline glioma patients: a survey of members of the AANS/CNS Tumor Section
Source: J Neurooncol. 2020 Jul 23;149(1):161–70. doi: 10.1007/s11060-020-03585-7 (PMC7452882; doi:10.1007/s11060-020-03585-7)

Variations in attitudes towards stereotactic biopsy of adult diffuse midline glioma patients: A survey of members of the AANS/CNS Tumor Section

Journal of Neuro-Oncology

John Lynes, MD, Alvina A. Acquaye, MS, Hannah Sur, BS, Anthony Nwankwo, BS, Victoria Sanchez, BS, Elizabeth Vera, MS, Tianxia Wu, PhD, Brett Theeler, MD, Terri Armstrong, PhD, Mark Gilbert, MD, Edjah K. Nduom, MD*

*Corresponding Author:
Edjah K. Nduom, M.D.
Surgical Neurology Branch
National Institute of Neurological Disorders and Stroke, NIH
10 Center Drive
Room 3D20
Bethesda, MD 20892
Email: edjah.nduom@nih.gov
Twitter: @EKNduom

Adult Diffuse Midline Glioma Survey N = 81

(% = # respondents that selected this answer / # respondents that answered this question)

1. What is your practice setting? n = 81

Answer	Responses
Academic center	67 (82.74%)
Subspecialized group practice, non-academic	6 (7.41%)
Small group practice (2-5 neurosurgeons)	6 (7.41%)
Solo practitioner	2 (2.47%)

2. When did you complete neurosurgical residency training? n = 71

Date

MM/DD/YYYY

Mean: 14.21 yrs
Range: 0.28 – 38.43 yrs

3. Do you participate in a multidisciplinary tumor board at your institution? n = 81

Answer	Responses
Yes, on a monthly basis	15 (18.52%)
Yes, on a weekly basis	59 (72.84%)
There is one, but I do not attend	2 (2.47%)
We do not have a tumor board at my institution	5 (6.17%)

4. How many adult (18 or older) glioma patients (any grade) did you operate on, in the past 12 months (biopsy or resection)? n = 80

Answer	Responses
10 or less	23 (28.75%)
11 to 25	20 (25%)
26- 50	20 (25%)
50 or more	17 (21.25%)

5. How many adult (18 or older) patients with diffuse midline gliomas (basal ganglia, thalamus, brainstem or spinal cord) did you see in the past 12 months? n = 81

Answer	Responses
0	7 (8.64%)
1-5	49 (60.49%)
6-10	18 (22.22%)
10+	7 (8.64%)

6. Are there any of the following brain locations where you would not routinely offer a biopsy for a diffuse glioma? (Please choose all that apply, or none, if you would routinely biopsy all midline locations) n = 81

Answer	Responses
Basal Ganglia	5 (6.17%)
Thalamus	4 (4.94%)
Brainstem	41 (50.62%)
Spinal cord	19 (23.46%)
None	33 (40.74%)

Adult Diffuse Midline Glioma Survey

Biopsy Not Offered (All locations)

7. Since you answered that one or more of the locations in Q.6 is one that you will not biopsy, why don't you offer biopsies in these locations? (Choose all that apply) n = 3

- [ ] It will not change the management for the patient
- [ ] The morbidity is too high in these locations — 3 (100%)
- [ ] I refer these patients to another center
- [ ] Not familiar enough with biopsies in this area
- [ ] Other (please specify)

8. From your responses to the previous question, please rank in order your choices from the most to least common reason that you would not biopsy a particular patient? (Use the following scale 1 (most common)-5 (least common)) n = 2

**number of respondents that ranked an answer as 1*

It will not change the management for the patient	
The morbidity is too high in these locations	2 (100%)
I refer these patients to another center	
Not familiar enough with biopsies in this area	
[Insert text from Other]	

9. Since you do not offer biopsies, what is the next step in care for these patients? n = 2

- ○ Referral directly to a radiation-oncologist for radiation — 1 (50%)
- ○ Referral directly to a neuro-oncologist/medical oncologist for chemotherapy
- ○ Referral to another center for diagnosis and treatment
- ○ Referral to both radiation and medical oncology for combination chemo-radiation — 1 (50%)
- ○ Other (please specify)

Biopsy Not Offered

10. Since you answered that one or more of the locations in Q.6 is one that you will not biopsy, why don't you offer biopsies in these locations? (Choose all that apply) n = 46

- [ ] It will not change the management for the patient — 17 (36.96%)
- [ ] The morbidity is too high in these locations — 32 (69.57%)
- [ ] I refer these patients to another center — 5 (10.87%)
- [ ] Not familiar enough with biopsies in this area — 2 (4.35%)
- [ ] Other (please specify) — 5 (10.87%)

11. From your responses to the previous question, please rank in order your choices from the most to least common reason that you would not biopsy a particular patient? (Use the following scale 1 (most common)-5 (least common)) n = 45

number of respondents that ranked an answer as 1

- It will not change the management for the patient — 11 (24.44%)
- The morbidity is too high in these locations — 26 (57.78%)
- I refer these patients to another center — 4 (8.89%)
- Not familiar enough with biopsies in this area — 1 (2.22%)
- [Insert text from Other] — 3 (6.67%)

12. Since you do not offer biopsies, what is the next step in care for these patients? n = 45

- ○ Referral directly to a radiation-oncologist for radiation — 3 (6.67%)
- ○ Referral directly to a neuro-oncologist/medical oncologist for chemotherapy — 1 (2.22%)
- ○ Referral to another center for diagnosis and treatment — 5 (11.11%)
- ○ Referral to both radiation and medical oncology for combination chemo-radiation — 36 (80%)
- ○ Other (please specify)

Biopsy Offered

13. Since you previously answered that you routinely offer biopsies for some or all diffuse midline gliomas, why do you offer them? n = 76

- [ ] My radiation oncologist will not treat these patients without tissue.
- [ ] To rule out a resectable lesion
- [ ] Research purposes (tissue banking)
- [ ] Clinical trial eligibility
- [ ] My medical oncologist will not treat these patients without tissue.
- [ ] Other (please specify)

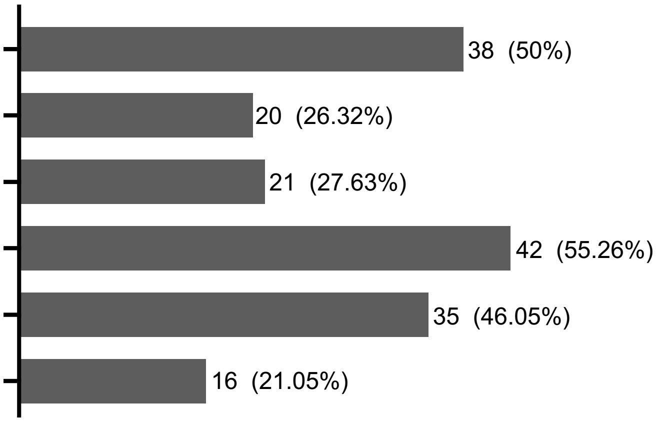

14. From your responses to the previous question, please rank in order your choices from the most to least common reason that you would offer biopsy to a particular patient? (Use the following scale 1 (most common)- 6 (least common)) n = 71

number of respondents that ranked an answer as 1

[ ]	My radiation oncologist will not treat these patients without tissue.
[ ]	To rule out a resectable lesion
[ ]	Research purposes (tissue banking)
[ ]	Clinical trial eligibility
[ ]	My medical oncologist will not treat these patients without tissue.
[ ]	[Insert text from Other]

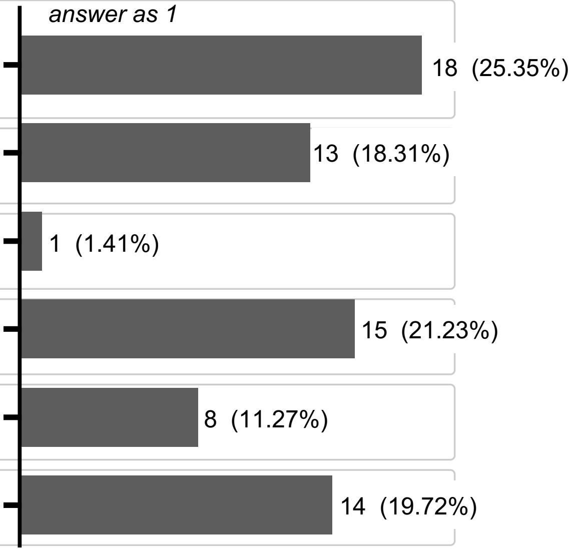

15. Since you do offer biopsies, what is done with the tissue? (Choose all that apply) n = 72

- [ ] Standard H&E pathological assessment to determine treatment
- [ ] Molecular analysis (mutations) to determine treatment
- [ ] Frozen tissue banking
- [ ] Next generation sequencing to determine treatment
- [ ] Other (please specify)

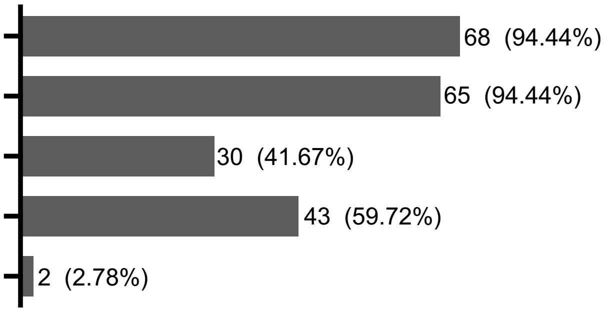

16. What would make you more likely to consider biopsies on adult diffuse midline glioma patients or referral of these patients to a center where they are performed? (Please check all that apply) n = 73

- [ ] Publications demonstrating safety and utility of the procedure
- [ ] New treatment options for these patients
- [ ] Clinical trials that require tissue for these patients
- [ ] Training on safe biopsy of these areas
- [ ] Other (please specify)

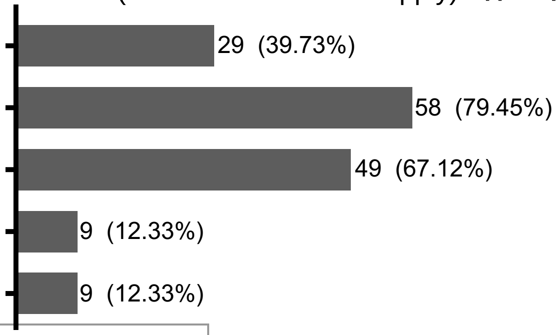

Supplement: Supplementary file 1 — Supplementary file1 (PDF 773 kb) [file 11060_2020_3585_MOESM1_ESM.pdf]
