# Supplementary material for: Variations in attitudes towards stereotactic biopsy of adult diffuse midline glioma patients: a survey of members of the AANS/CNS Tumor Section
Source: J Neurooncol. 2020 Jul 23;149(1):161–70. doi: 10.1007/s11060-020-03585-7 (PMC7452882; doi:10.1007/s11060-020-03585-7)
Supplement: Supplementary file 2 — Supplementary file2 (DOCX 28 kb) [file 11060_2020_3585_MOESM2_ESM.docx]

**Checklist adopted from “Good practice in the conduct and reporting of survey research”**

Kate Kelley, Belinda Clark, Vivienne Brown, John Sitzia*. International Journal for Quality in Health Care*, Volume 15, Issue 3, May 2003, Pages 261–266, https://doi.org/10.1093/intqhc/mzg031

When reporting survey research, it is essential that a number of key points are covered (though the length and depth of reporting will be dependent upon journal style). These key points are presented as a ‘checklist’ below:

1. **Explain the purpose or aim of the research, with the explicit identification of the research question.**

*This is addressed in the Objective of the study, in the Abstract:*

“Our goal was to assess the attitudes of current practicing neurosurgical oncologists towards management of adult diffuse midline gliomas, reasons behind their practices, and factors that might influence these practices.”

1. **Explain why the research was necessary and place the study in context, drawing upon previous work in relevant fields (the literature review).**

*This is addressed in the introduction. In part:*

*“*Recent advancements in the field have discovered relevant molecular mutations in DMGs ^13,14^, giving way to broad developments in diagnosis and treatment of these tumors and reason to biopsy these patients to develop targeted therapeutics ^15^ and clinical trials to test them. However, despite this progress, the standard practice for diagnosing and treating this disease is unclear and management can vary across clinical sites. We sought to describe the current range of practice by practicing neurosurgeons. Our survey assessed the attitudes of neurosurgical oncologists who are members of the Joint Tumor Section of the American Association of Neurological Surgeons (AANS) and Congress of Neurological Surgeons (CNS) regarding tissue sampling of DMGs. Our aim was to define their current decision making in the management of diffuse midline gliomas, reasoning for their practices, and potential factors that may influence or change their practice.”

1. **Describe in (proportionate) detail how the research was done.**
   1. **State the chosen research method or methods, and justify why this method was chosen.**
   2. **state its psychometric properties and provide references to the original development work. If a new tool is used, you should include an entire section describing the steps undertaken to develop and test the tool, including results of psychometric testing.**
   3. **Describe how the sample was selected and how data were collected, including:**
   4. **How were potential subjects identified?**
   5. **How many and what type of attempts were made to contact subjects?**
   6. **Who approached potential subjects?**
   7. **Where were potential subjects approached?**
   8. **How was informed consent obtained?**

*The above are addressed in the Methods, as follows:*

“An open and voluntary survey consisting of 16 multiple-choice questions (see Supplemental Digital Content for full survey) was developed by a multi-disciplinary group of expert providers, including representative from the Surgical Neurology Branch (SNB) of NINDS and the NCI-CONNECT program of the Neuro-Oncology Branch (NOB), CCR, NCI, NIH. It was also vetted by the Tumor Section Survey Committee prior to approval. This research survey was excluded from IRB Review per 45 CFR 46 and NIH policy (OHSRP ID#: 19-NINDS-00813) for the use of survey procedures because it involved the use of survey procedures and the recorded information was not linked to identifiable human subjects, directly or through identifiers linked to the subjects. The survey was web-based and sent via an e-mailed link to the 636 active members of the Joint Tumor Section of the AANS and CNS list serve and advertised via the official Twitter account of the Joint Tumor Section (@NSTumorSection). The Tumor Section is a voluntary, membership-only group of practicing neurosurgeons who specialize in tumors of the central nervous system. Responses were collected for 4 weeks (from 10/2/19 to 10/30/19). It was created on Surveymonkey.com with allowance for custom answers to specific questions for respondents if they believed the choices provided did not accurately reflect their response. As some questions depended on answers to previous questions, the order of the questions were not randomized and adaptive questioning was employed. Any user with the survey link was free to participate; no incentives were offered. All of the answers were automatically captured by the secure SurveyMonkey database and exported to an excel and PDF file for analysis. To prevent multiple entries from the same individual, IP addresses were collected and duplicate entries were ‘turned off’ for the survey, thus only allowing one survey response per device. These IP addresses could not be traced to an individual person, but rather to a single device, proxy server, or group of devices on the same network. Responses for each question were grouped based on potential significance and/or frequency that a particular answer was chosen. These groups were then compared to surgeon and institution-specific characteristics in order to assess for correlations.”

- 1. **How many agreed to participate?**
  2. **How did those who agreed differ from those who did not agree?**
  3. **What was the response rate?**

*These questions are addressed in the Results section in detail. In part, this reads:*

“A total of 81 neurosurgeons completed the survey out of 636 active members of the Tumor Section of CNS. However, a wide range in the number of responses were submitted for each of the 16 questions (range 2-81), as answering all questions was not required for submission and the survey logic presented different questions based off of answers to previous questions from question 7 onwards (Supplemental Digital Content). Respondents included surgeons with varied training backgrounds and practices, which provided a broad view of surgeon and institution-specific characteristics as well as management strategies. A large majority (83%) of respondents practice in academic centers and 91% participate in tumor boards at their institutions (Figure 2). 90% of surgeons had some form of molecular analysis available at their institutions, though more advanced next generation sequencing was only available to 60% of these. Surgeons ranged widely in their time in practice, from approximately 4 months to over 38 years with a mean of 14.21 years.”

1. **Describe and justify the methods and tests used for data analysis.**

*Our data analysis is described in the Methods:*

*“Statistical Analysis*

A one-way analysis of variance (ANOVA) was performed to evaluate differences in years of experience between surgeons who offered and did not offer biopsies for a diffuse glioma (questionnaire 6 ,10, 11,13, 14 and 16). The experience years were calculated from the date neurosurgical residency training was completed to the date the survey was completed. Fisher’s exact test was applied to test the association between the number of operated patients in the past 12 months (Q4) and the willingness to offer biopsy at various midline locations (Q6, Q10, Q11, Q13, Q14, Q16). The number of operated patients was dichotomized into a binary variable: ≤25 and >25.  Fisher’s exact test was also applied to evaluate the association between the reason of offering biopsy (Q13) and treatment decision (Q15). SAS software 9.4 was used for statistical analyses. A statistical significance level was set at α = 0.05.

*Qualitative Analysis*

Given that four of the questions (Q10, Q13, Q15, Q16) included the ability to answer “Other” and give a personalized response, qualitative analysis of these responses was performed using MAXQDA 2019 software (VERBI Software, Berlin, Germany). Responses were then categorized into parent codes and direct subcodes. Additionally, for these four questions, word clouds were created from survey respondent answers (Figure 1A-C).”

1. Present the results of the research. The results section should be clear, factual, and concise.

*We believe that our results section fits these guidelines*

1. Interpret and discuss the findings. This ‘discussion’ section should not simply reiterate results; it should provide the author’s critical reflection upon both the results and the processes of data collection. The discussion should assess how well the study met the research question, should describe the problems encountered in the research, and should honestly judge the limitations of the work.

*We believe our discussion section places the survey in appropriate context, as requested here.*

1. Present conclusions and recommendations.

*We limited our conclusions to the data presented. Our recommendations are presented at the end of the discussion.*
